# A WeChat Mini Program-Based Intervention for Physical Activity, Fruit and Vegetable Consumption Among Chinese Cardiovascular Patients in Home-Based Rehabilitation: A Study Protocol

**DOI:** 10.3389/fpubh.2022.739100

**Published:** 2022-03-22

**Authors:** Yanping Duan, Xin Li, Lan Guo, Wei Liang, Borui Shang, Sonia Lippke

**Affiliations:** ^1^Department of Sport, Physical Education and Health, Faculty of Social Sciences, Hong Kong Baptist University, Kowloon, Hong Kong SAR, China; ^2^Centre for Health and Exercise Science Research, Hong Kong Baptist University, Kowloon, Hong Kong SAR, China; ^3^Department of Physical Education, School of Physical Education (Main Campus), Zhengzhou University, Zhengzhou, China; ^4^Cardiac Rehabilitation Center, Department of Cardiology, Guangdong Provincial People's Hospital, Guangzhou, China; ^5^Department of Social Sciences, Hebei Sport University, Shijiazhuang, China; ^6^Department of Psychology and Methods, Jacobs University Bremen, Bremen, Germany

**Keywords:** WeChat mini program-based intervention, health action process approach, physical activity, fruit and vegetable consumption, cardiovascular diseases, home-based rehabilitation

## Abstract

**Background:**

Cardiac rehabilitation programs aim to avoid further progression and relapse of cardiovascular diseases (CVDs). Patients with CVDs undergoing rehabilitation often experience difficulties in integrating and transferring the learned health behaviors into their daily life after returning home. This includes regular physical activity (PA) and sufficient fruit and vegetable consumption (FVC). eHealth individualized interventions have shown to be effective in increasing PA and FVC in home settings. As WeChat is the most popular social media site in China, this should be used for the intervention. The aim of this study is to develop and examine a WeChat mini program-based intervention on PA and FVC promotion among Chinese cardiovascular patients in home-based rehabilitation.

**Methods:**

The study will adopt a randomized controlled trial (RCT), comprising a WeChat mini program-based intervention group and a waiting-list control group. The intervention content will be designed based on the Health Action Process Approach (HAPA). One hundred fifty-eight outpatients will be recruited from the cardiac rehabilitation center of a hospital in southern China and randomly assigned to one of the two groups. During the 10-week duration of the intervention, participants will be invited to access a WeChat mini program comprising two Modules. Module 1 provides weekly learning sessions addressing PA and FVC simultaneously for 10 weeks. Module 2 provides a platform, where participants can review their progress with Module 1 at any time and attend incentive activities aiming at promoting engagement and retention. The outcome variables include PA (mins/week), FVC (portion/day), healthy lifestyle (the synthesis of PA and FVC), social-cognitive predictors of behavior change (risk perception, outcome expectancies, self-efficacy, intention, planning, social support, and action control) as well as health outcomes (Body mass index, depression, and quality of life). Data collection will be implemented at pre-test, post-test and a post-test after 3-month respectively.

**Discussion:**

The current study will be significant to understand how such a cost-effective social media mini program-based intervention enables participants to adopt and maintain a healthy lifestyle. If it is effective, it will enrich home-based cardiac rehabilitation approaches which can in turn save the lives of patients as well as much monetary, time and other investments.

**Trial Registration:**

The study was registered at ClinicalTrials.gov (Identifier: NCT03636724; Last update posted: July 28, 2020).

## Introduction

Cardiovascular diseases (CVDs), the leading cause of death worldwide (1), are a group of disorders affecting the heart and blood vessels. These diseases include coronary heart disease, cerebrovascular disease, peripheral arterial disease etc. (2). In China, it was estimated that 290 million people suffered from CVDs, causing 40% of the annual total deaths (3). As an indispensable component in the CVD recovery process, cardiac rehabilitation has been proved to be effective for the prevention of further progression and relapse of CVDs (4). Cardiovascular patients receive the guidance provided on healthy lifestyle including regular physical activity (PA) and a healthy diet during rehabilitation in their hospitalization (5). However, the significant contribution of rehabilitation mainly depends on its successful implementation in daily life after the formal rehabilitation process has ended. Therefore, home-based rehabilitation can extend the benefits obtained from facilities before and motivate patients with maintaining a healthy lifestyle in daily life (6, 7). In China, however, some home-based rehabilitation aftercare offers (e.g., telephone consultation, household survey) are inaccessible to patients, especially those population who live in small-sized cities and remote areas (8). In addition, home-based rehabilitation is not well researched as it employs the self-management of the patients over longer periods of time.

PA has been shown to be beneficial for the rehabilitation of cardiovascular patients. By regularly participating in PA (i.e., at least 150 min weekly of moderate PA), rehabilitation patients with CVDs can enhance their physical and mental condition such as reduced blood pressure, better blood lipids and cholesterols, and relieved stress (9, 10). On the other hand, as an important component of a healthy diet, fruits and vegetables are rich sources of vitamins and minerals, dietary fiber, and a host of beneficial antioxidants. The significant association between sufficient FVC (i.e., at least five servings daily) and reduced CVD-related symptoms has been proved among cardiovascular patients in previous research (11, 12).

As an emerging delivery channel for health services using the internet and related technologies and media (e.g., computers, smart phones/watches), eHealth approaches have been widely applied to disease prevention and management this decade (13). The effectiveness of eHealth interventions on PA and FVC has been proven to facilitate healthy lifestyle changes after discharge among patients with CVDs (14–16). In China, most eHealth home-based cardiac rehabilitation interventions merely focus on knowledge, education, and telephone consultation. Very few integrate individualized and comprehensive interventions that include educational, cognitive, and psychological elements (5, 17, 18).

This study will apply the Health Action Process Approach (HAPA) (19) as the theoretical underpinning for the PA and FCV interventions. The HAPA model postulates that behavior change is a dynamic and continuous process, which comprises two distinctive phases. The first phase is the motivational phase, which plays an important role to increase risk perception (e.g., risk likelihood of suffering from a heart attack), action self-efficacy (e.g., how to stay confident about the ability to sufficiently perform FVC), and positive outcome expectancies (e.g., expected pros of the outcomes of participating in PA) (20); this will strengthen the formation of intentions for behavior change (e.g., “I intend to do physical activities at least 5 days per week with 30 min each time” or “I intend to eat five portions of fruits and vegetables per day”).

Once individuals have established an intention, they enter the second phase namely the volitional phase. In this phase, individuals may benefit from a series of volitional self-regulation strategies for behavior initiation and maintenance (16). This includes action planning (e.g., when, where, and how to enact PA), coping planning (e.g., how to maintain PA levels when confronted with barriers), maintenance self-efficacy (e.g., how to stay confident about the ability to eat sufficient portions of fruit and vegetable when obstacles occur), recovery self-efficacy (e.g., how to stay confident about the ability to re-start PA behavior after the disengagement), and action control (how to constantly self-monitor themselves to prevent relapse). In addition, promoting individual's perceived social support is equally important in maintaining behavior and preventing relapse (19, 21).

In a previous study (18), the effects of a HAPA-based, internet-delivered intervention for PA and FVC changes in patients with coronary heart disease in home-based rehabilitation were examined. It was found that the intervention outperformed the control condition for PA, FVC, psychological resources of behaviors (e.g., self-efficacy and planning of PA and FVC, social support of FVC) and health outcomes (e.g., quality of life). The results with an eta^2^ effect size ranging from 0.06 to 0.43 underscore the usefulness. In addition, the intervention effect was seen with a higher percentage of healthy lifestyle adoption (the synthesis of PA and FVC) (40% intervention group vs. 10% control group) (18). However, only a pre-test and post-test were measured in this study with a small sample size (*n* = 83). Furthermore, this study was only based on some parts of the HAPA constructs, where the action control which plays a crucial role in the behavioral maintenance (19), has not been included in the intervention content. In addition, the intervention duration for each health behavior (PA/FVC) was only 4 weeks and the study just included patients with access to the internet *via* a computer.

WeChat, a popular social media site with over 800 million active users, similar to Facebook and Twitter, is widely used in China. It has great potential for cardiac rehabilitation intervention (22). WeChat mini programs are similar to general apps, however, there is no need to install or uninstall them on the smartphone. This dramatically improves accessibility and convenience (23) and with that the likelihood that it is used extensively. Until now, WeChat mini programs focusing on healthy lifestyle intervention in rehabilitation patients with CVD in China or elsewhere are still scarce.

To address the aforementioned research gap and the limitation of our previous study, the aim of the present study is to develop, implement, and evaluate a 10-week WeChat mini program-based PA and FVC intervention for Chinese patients with CVD in home-based rehabilitation. Four hypotheses will be tested in this study: (1) participants in the intervention group will have more behavioral change in PA and FVC compared to the control group; (2) there will be more adoption of a healthy lifestyle (the synthesis of PA and FVC) in the intervention group compared to the control group; (3) participants in the intervention group will have more improvements in social-cognitive predictors of behavior change compared to the control group; (4) participants in the intervention group will have more improvements in health outcomes (BMI, depression level and quality of life) compared to the control group.

## Methods and Analysis

### Design

The current study will be a randomized controlled trial (RCT) comprising two groups: a WeChat mini program-based intervention group (IG) and a waiting-list control group (WLCG). The IG will address PA and FVC intervention simultaneously for 10 weeks while the WLCG will get access to the intervention after the completion of all data collection for the IG. Patients in the two groups will attend data collection three times. The pre-test will take place after randomization and before intervention start or the waiting time (T1). After the completion of the 10-week intervention of the IG, all patients will be invited to complete the post-test immediately (T2) and a subsequent follow-up assessment at 3 months after the post-test (T3). This study has no potential adverse effects. It received ethics approval from the Research Ethics Committee (REC) of Hong Kong Baptist University (Ref. No.: FRG2/17-18/099) and was registered with ClinicalTrials.gov (Identifier: NCT03636724).

### Selection/Treatment of Subjects

The target participation will be outpatients with CVDs. For the inclusion criteria, eligible participants should (1) be aged between 18 and 75 years; (2) have no restriction of physical mobility under the cardiac function at entry; (3) have no restriction of other diseases such as diabetes or fruit and vegetable allergies/ intolerance; (4) have no cognition-related disorders or mental diseases; (5) have passed the Physical Activity Readiness Questionnaire screening or obtained the physician's approval prior to the participation; (6) do not attend other ongoing programs involving healthy lifestyle promotion (physical exercise and/or nutrition); (7) have sufficient reading and writing language skills in Chinese; and (8) be able to access WeChat and the internet *via* a smartphone.

The sample size was calculated by using G^*^Power 3.1 software with MANOVA with repeated measures (24). For achieving a medium effect size (Cohen's f 0.3) on PA and FVC respectively based on our previous pilot study (18), with a statistical power (1-β) of 0.8 and alpha of 0.05, the total sample size is estimated as *n* = 111. Based on the findings of our previous study with similar samples (18) and to ensure a robust statistical power for the effects estimate (25), we assume a 30% dropout rate of participants; therefore, a total of 158 participants will be required for the current study, with 79 participants for each group.

**Figure 1:**
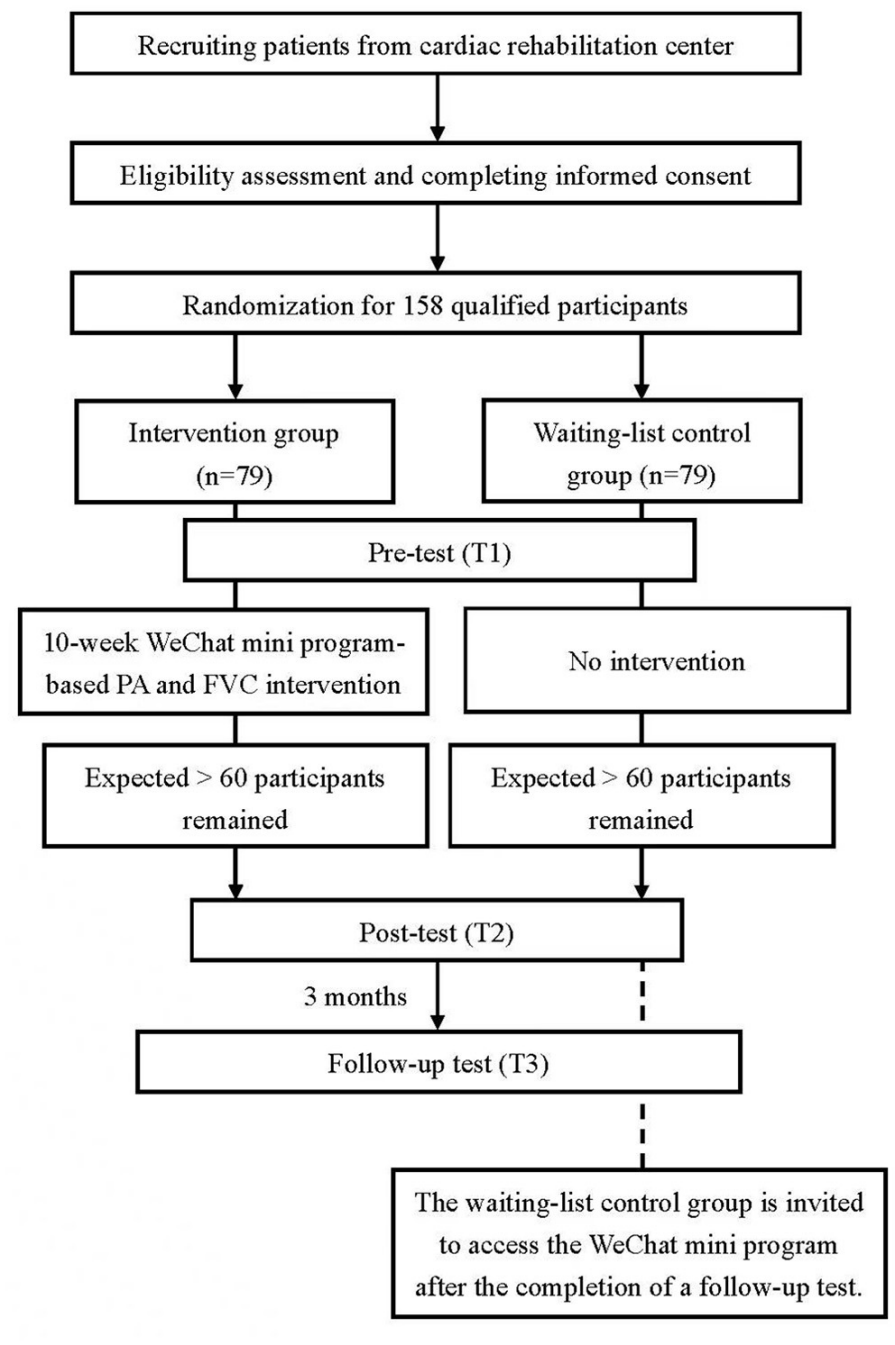
Flowchart of patients' progress throughout the study phases. PA, Physical Activity; FVC, Fruit and Vegetable Consumption.

Outpatients with CVDs will be recruited face-to-face by the physician of the research team, with the assistance of two research nurses at the Department of Cardiology in Guangdong Provincial People's Hospital, Guangzhou, China. The cardiac rehabilitation center in the Department of Cardiology is one of the largest cardiac rehabilitation centers in southern China, where more than 4,000 patients with CVDs were treated each year (26). The recruitment will last for five and a half months based on our previous study experience to ensure us to recruit sufficient participants (18). After the eligibility check, patients who express interest in participating in the study and sign the informed consent form will be invited to complete the online registration *via* our WeChat mini program. Then all the qualified patients will be allocated with equal probability (1:1 ratio) to the intervention group and control group. The randomization will be conducted by project researchers on the backend system of the mini program using a simple randomization list. Figure 1 shows the flowchart of patients from recruitment to allocation in the study.

### Interventional Development

The intervention development will be facilitated through four steps. Step 1: Research team members will develop the intervention content of 10-week sessions including PA and FVC. Step 2: A WeChat mini program will be established by an Internet Technology Company based on the intervention content. Step 3: Four outpatients with CVDs (two females and two males) will be invited to pilot test the function of WeChat mini program. Step 4: Amendment and refinement will be made for the WeChat mini program based on the suggestions of participants in the pilot test.

#### Intervention Group

A WeChat mini program namely e-health homeland (in Chinese: 健康家园网站) will be established to facilitate intervention. The mini program will comprise two modules. Module 1 consists of a 10-week intervention program targeting HAPA-based social-cognitive predictors of PA and FVC. Patients will be invited to access this module once per week. Module 2 provides a platform for data repository, collection of former exercises and incentive activities, where patients can access all their files at any time during the 10 weeks of intervention.

In particular, for Module 1, the targeted social-cognitive variables for PA and FVC in 10 weeks are as follows: week 1—risk perception and outcome expectancies; week 2—action self-efficacy and goal setting; week 3—action self-efficacy and action planning; week 4—maintenance self-efficacy and action planning; week 5—maintenance self-efficacy, action planning and coping planning; week 6—maintenance self-efficacy and coping planning; week 7 and 8—maintenance self-efficacy, recovery self-efficacy, coping planning and social support; week 9 and 10—maintenance self-efficacy, recovery self- efficacy, coping planning, social support and action control. In order to facilitate the behavior initiation and maintenance, a series of behavior change techniques (BCTs) will be employed in the intervention such as informing about the risks and negative consequences of unhealthy behaviors [BCT 5.1–5.6], teaching about the benefits of healthy behaviors [BCT 5.1–5.6], prompting verbal persuasion about the participant's capability of performing and maintaining healthy behaviors [BCT 15.1–15.2], motivating to set specific behavioral goals [BCT 1.1], training patients to make action plans [BCT 1.4], identifying barriers of behavior performance and making coping plans [BCT 1.2], evaluating and adjusting action plans and coping plans [BCT 1.2, 1.4, 1.6], and prompting self-monitoring health behaviors [BCT 2.1–2.7] (27). The intervention targeting variables and BCTs for each week are shown in Table 1.

**Table 1:** Intervention targeting variables and behavior change techniques for each week.

**Week**	**Intervention targeting variables**	**Behavior change techniques [BCT, (** **27** **)]**	
		**PA**	**FVC**
Week 1	Risk perception Outcome expectancies	1) Informing about the risks and negative consequences of sedentary behavior and insufficient PA. 2) Informing about the health benefits of PA.	1) Informing about the risks and negative consequences of insufficient FVC. 2) Informing about the health benefits of FVC.
Week 2	Action self-efficacy Goal setting	1) Prompting verbal persuasion about capability of performing PA; Providing example of successful case about PA participation. 2) Asking patients to set goals for PA.	1) Prompting verbal persuasion about capability of FVC; Providing example of successful case about FVC. 2) Asking patients to set goals for FVC.
Week 3	Action self-efficacy Action planning	1) Prompting verbal persuasion about capability of performing PA; Providing example of successful case about PA participation. 2) Asking patients to make specific action plans of when, where and how to perform PA.	1) Prompting verbal persuasion about capability of FVC; Providing example of successful case about FVC. 2) Asking patients to make specific action plans of when, where and how to eat fruit and vegetables.
Week 4	Maintenance self-efficacy Action planning	1) Prompting verbal persuasion about capability of maintaining PA; Providing example of successful case about PA adherence. 2) Asking patients to evaluate their execution of PA action plans and adjust PA action plans if necessary.	1) Prompting verbal persuasion about capability of maintaining FVC; Providing examples of successful cases about FVC adherence. 2) Asking patients to evaluate their execution of FVC action plans and adjust FVC action plans if necessary.
Week 5	Maintenance self-efficacy Action planning Coping planning	1) Prompting verbal persuasion about capability of maintaining PA; Providing example of successful case about PA adherence. 2) Asking patients to evaluate their execution of PA action plans and adjust PA action plans if necessary. 3) Asking patients to identify their barriers to PA execution and make coping plans.	1) Prompting verbal persuasion about capability of maintaining FVC; Providing example of successful case about FVC adherence. 2) Asking patients to evaluate their execution of FVC action plans and adjust FVC action plans if necessary. 3) Asking patients to identify their barriers to FVC execution and make coping plans.
Week 6 (Boosting session)*	Maintenance self-efficacy Coping planning	1) Prompting verbal persuasion about capability of maintaining PA; Providing example of successful case about PA adherence. 2) Asking patients to evaluate their execution of PA coping plans and adjust PA coping plans if necessary.	1) Prompting verbal persuasion about capability of maintaining FVC; Providing example of successful case about FVC adherence. 2) Asking patients to evaluate their execution of FVC coping plans and adjust FVC coping plans if necessary.
Week 7 and Week 8	Maintenance and recovery self-efficacies Coping planning Social support	1) Prompting verbal persuasion about capability of maintaining PA; Providing example of successful case about PA adherence and relapse prevention. 2) Asking patients to evaluate their execution of PA coping plans and adjust PA coping plans if necessary. 3) Asking patients to review their perceived social support on PA from significant others; Providing example of successful case about having support on PA participation.	1) Prompting verbal persuasion about capability of maintaining FVC; Providing example of successful case about FVC adherence and relapse prevention. 2) Asking patients to evaluate their execution of FVC coping plans and adjust FVC coping plans if necessary. 3) Asking patients to review their perceived social support on FVC from significant others; Providing example of successful case about having support on FVC.
Week 9 and Week 10	Maintenance and recovery self-efficacies Coping planning Social support Action control	1) Prompting verbal persuasion about capability of maintaining PA; Providing example of successful case about PA adherence and relapse prevention as a model for the patient. 2) Asking patients to evaluate their execution of PA coping plans and adjust PA coping plans if necessary. 3) Asking patients to review their perceived social support on PA from significant others; Providing example of successful case about having support on PA participation. 4) Guiding patients to write a reflective diary of PA participation within the past week (e.g., date, time, PA type, venue, emotional experience) to record and self-monitor their PA.	1) Prompting verbal persuasion about capability of maintaining FVC; Providing example of successful case about FVC adherence and relapse prevention as model for the patient. 2) Asking patients to evaluate their execution of FVC coping plans and adjust FVC coping plans if necessary. 3) Asking patients to review their perceived social support on FVC from significant others; Providing example of successful case about having support on FVC. 4) Guiding patients to write a reflective diary of FVC in a recent day (e.g., date, time, FV type, venue, emotional experience) to record and self-monitor their FVC.

*PA, physical activity; FVC, fruit and vegetable consumption*.

* *Project researchers will give a phone call to patients in order to boost them to engage in and adhere to recommended PA and FVC level*.

**Figure 2:**
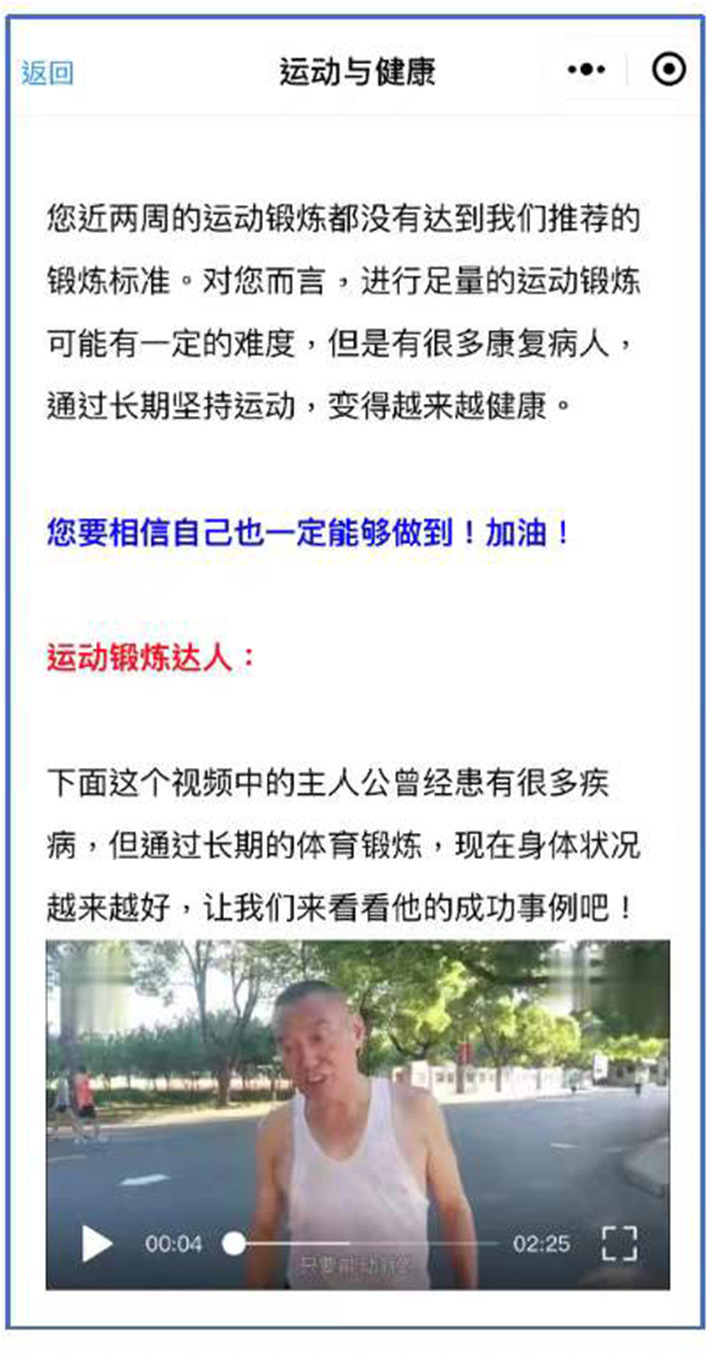
A text and video screenshot of a role model telling his story about gaining health benefits by engaging in jogging for several years.

In addition, patients will receive tailored individualized, automated feedback on their PA and FVC behaviors at the beginning of each session, based on their prior self-report questionnaire. The examples of the feedback can be found in our previous publication (28). Moreover, examples of role models [BCT 6.2] will be provided throughout the intervention to support patients to increase action self-efficacy, maintenance self-efficacy and recovery self-efficacy, develop plans, and improve social support. The examples can be displayed in the form of text and photos/short videos. Figure 2 shows a text and video screenshot of a role model with coronary heart disease, who shares his story about gaining health benefits by engaging in jogging for many years. Furthermore, in each week's session, patients can select either PA or FVC as the firstly addressed behavior according to their personal preference. The weekly intervention will last for about 20 min.

**Figure 3:**
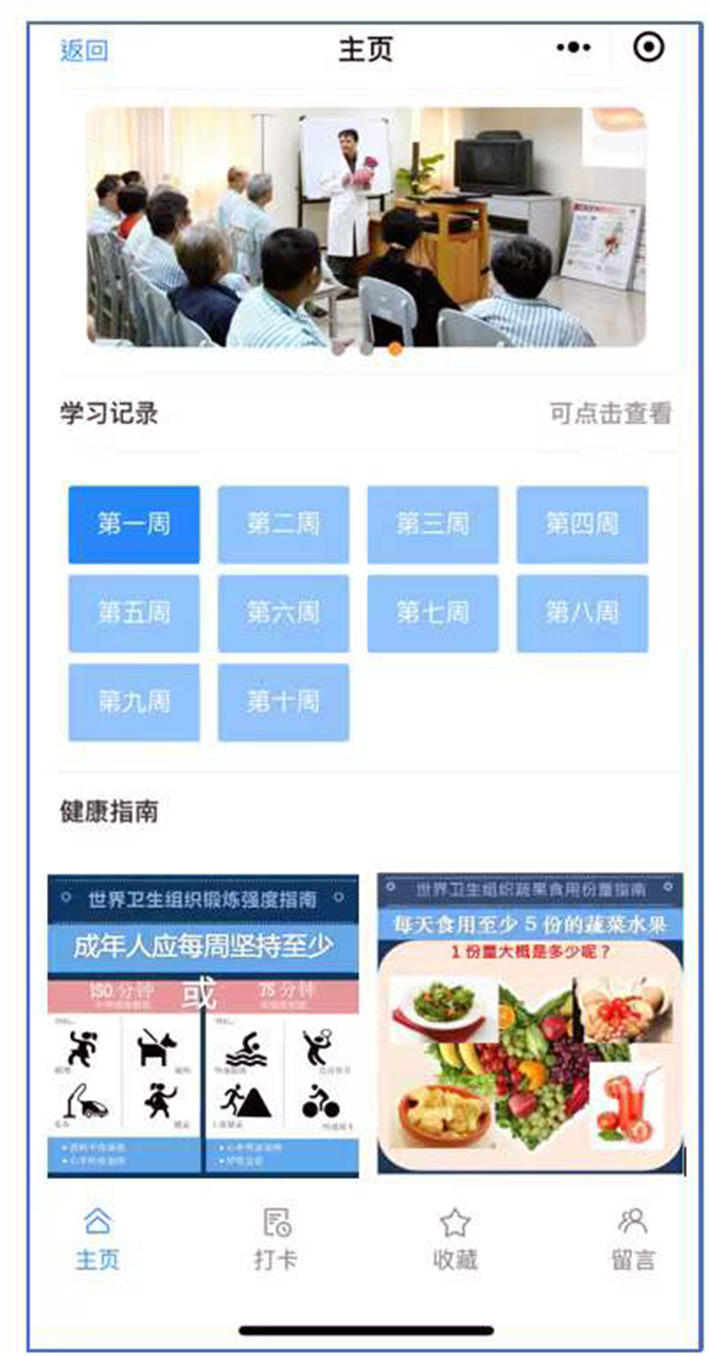
The layout screenshot of the home page in Module 2. The four Chinese characters at the last row of the screenshot from left to right stand for four sections of Module 2: home page, check-in, archive and open forum.

In addition to Module 1, Module 2 will consist of four sections, including Home Page, Check-In, Archive, and Open Forum. In the Home Page section, patients can review each session content they completed in Module 1 (see Figure 3). In the Archive Section, patients can review their data aggregated in Module 1 under four sub-sections including “*My Behavior Record*” (graphs of individualized feedback on PA and FVC in each week), “*My Action Plans*” (completed action plans for PA and FVC), “*My Coping Plans*” (completed coping plans for PA and FVC) and “*My Diary*” (completed reflective diaries for PA and FVC).

The Check-In section and Open Forum section will be open starting from Week 4 with the purpose of promoting participant engagement and retention. In particular, patients will be invited to check in once per day and indicate whether they have implemented PA and FVC each day accordingly. Monetary compensation in form of 2 RMB will be awarded to patients who complete the check-in with at least five times for PA and FVC respectively in each week. In addition, patients will be invited to share their PA and FVC experiences at the open forum and to have interactive communication with other patients and research nurses. Five RMB will be awarded to patients who rank the top three of the most active communicators at the forum per week. Furthermore, patients will be invited to write down their PA diary and FVC diary in the My Diary sub-section under the Archive at any time starting from Week 9. Additionally, 3 RMB will be awarded to patients who complete at least three diaries for PA/FVC each week. In addition, the WeChat message will be conveyed to the patients 1 day before each weekly session to remind them to attend the intervention accordingly. The participation time, frequency, and duration of each learning activity in Module 1 and 2 will be automatically recorded by the backend system.

#### Waiting-List Control Group

The control group will not receive any intervention during the 10-week study period. Participants in the control condition will be invited to access the WeChat mini program once the follow-up test is completed. With that, a waiting-list control group is achieved.

In addition, to monitor lifestyle correlates, participants in the intervention group and control group will record their medication used, sleep quality, illness situation and participation in other health-related activities (e.g., attending health workshops) by writing a logbook once per week. The research assistant will give WeChat calls to the participants twice a week, aiming to check their diaries and identify whether they have been confronted with adverse events (e.g., suffering acute illness). Data from those who had made a dramatic change in their normal lifestyles and health-related aspects will not be included in the subsequent data analysis.

### Measurements

All variables will be measured three times: pre-test (T1), post-test (T2), and after 3-month with a follow-up post-test (T3). If not mentioned otherwise, all items will be measured with the use of visual analog scales (VAS). VAS have been suggested to be better than Likert-scales, because of its user-friendly appearance and design, more accurate responses, and lower dropout rate (29).

#### Single Behavioral Indicators

The level of PA will be measured using an adaption of the Chinese short version of the International Physical Activity Questionnaire (30). Participants will be asked to indicate the number of days and time spent for vigorous, moderate and walking activities during the past week, with items such as “During the past 7 days, how many days did you do vigorous physical activities like fast bicycling, intensive swimming …”. The total PA score (in minutes/week) for each patient will be obtained by summing up all questions.

Daily FVC will be measured with four items incorporating “raw vegetables”, “fruit”, “fruit or vegetable juice”, and “cooked or steamed vegetables” followed by stems of counting the servings for each type of FVC (18, 31), such as “last week, how many portions of fruit did you eat per day”? Participants will be asked to count the number of portions of fruits and vegetables they consumed on average during a typical day. The total serving of daily FVC will be the sum of each relevant item.

#### Integrated Healthy Lifestyle Indicator

After obtaining the results of PA and FVC, participants will be categorized into one of two groups depending on whether they meet both PA and FVC recommendations (coding healthy lifestyle as 1) or not (coding unhealthy lifestyle as 0). The thresholds will be set according to the World Health Organization (WHO) recommendations, namely for moderate and vigorous PA at least 150 min/week, and for FVC at least five servings/day (18, 32).

#### Social-Cognitive Variables of Behavior Change

##### Risk Perception

Participants' risk perception will be measured using an adapted scale from Perloff and Fetzer (33). Items start with the stem: “How likely is it that you will have sometime in your life….”, followed by five items: “…a high cholesterol level?”, “…a heart attack?”, “…high blood pressure?”, “…a stroke?” and “…a cardiovascular disease?”. Answers can be given on a VAS-scale from very unlikely to very likely.

##### Outcome Expectancies

Participants' outcome expectancies for PA and FVC will be measured by four items respectively: two about positive and two about negative expectancies (16). For PA, questions will be asked with the stem “If I am physically active 5 days a week for at least 30 min, then…”, followed by four items such as “…this is good for my health”, and “…this will be a financial burden”. Regarding the FVC, outcome expectancies will be asked with the stem “If I eat five portions of fruits and vegetables daily, then…” followed by four items such as “…this is good for my health”, and “…this will be a financial burden”. Answers can be given on a VAS scale, ranging from totally disagree to totally agree.

##### Self-Efficacy

Self-efficacy will be measured in three parts including (1) motivational self-efficacy referring to one's confidence to perform a specific behavior; (2) maintenance self-efficacy referring to one's confidence to perform a specific behavior over a long period of time; and (3) recovery self-efficacy referring to one's confidence to resume a specific behavior after discontinuation (19).

Motivational self-efficacy will be assessed with one item for PA: “I am certain that I can be physically active a minimum of 5 days a week for 30 min even if it is difficult”. Additionally, there will also be one item to assess fruit and vegetable consumption: “I am certain that I can eat at least five portions of fruit and vegetables a day even if it is difficult” (31, 34).

Maintenance self-efficacy will be assessed with two items for each target behavior: “I am certain that I can be physically active regularly at a minimum of 5 days a week for 30 min … / “I am certain that I can regularly eat five portions of fruit and vegetable a day…” “… even if it takes a lot of time till I am used to do it” and “… even if I have worries and problems” (31, 34).

Recovery self-efficacy will be measured with two items for each behavior: “I am certain that I can again be physically active on at least 5 days a week for 30 min / I am certain that I can again eat five portions of fruit and vegetables a day …” “even if I changed my concrete plans several times” and “… even if I skipped a few times” (31, 34). Participants can answer all self-efficacy items on a VAS-scale ranging from don't agree at all to agree completely.

##### Intention

Intention for PA will be assessed with the stem “I intend to perform … at least 30 min a day for at least 5 days a week (or at least 150 min a week)”, followed by three items such as “… vigorous physical activity”, “… moderate physical activity”, and “… mild physical activity (i.e., walking)”. Regarding FVC, the intention will be assessed with the stem “I intend to …” followed by three items such as “…eat at least five portions of fruit and vegetables a day” (18, 28). Answers can be given on a VAS scale ranging from not true to exactly true.

##### Planning

Planning will be measured in two parts including action planning and coping planning. Action planning will be assessed by the stem “For the next month I have carefully planned…” followed by three items for PA such as “… where I will be physically active” or followed by three items for FVC such as “… at which meals I will eat fruit and vegetables”. Coping planning will be assessed by the stem “For the next month I have carefully planned…” followed by three items for PA such as “… how I continue to stay active even when something comes in between” or followed by three items for FVC such as “…how I continue to eat healthy even when something comes in between” (18, 28, 34). Answers can be given on a VAS-scale ranging from not agree at all to agree completely.

##### Social Support

Perceived social support will be assessed with three items for PA and FVC respectively such as “My partner helps me/my family helps me/my friends and acquaintances help me to stay physically active” or “My partner helps me/my family helps me/my friends and acquaintances help me to eat healthy” (18). Answers can be given on a VAS scale ranging from not agree at all to agree completely.

##### Action Control

Participants' action control will be measured using the scale of Sniehotta, Schulz, and Schwarzer (35). It consists of six items for PA and FVC respectively. Participants will be asked how much effort they exert in regulating their PA and FVC such as, “I have constantly monitored myself whether I exercise frequently enough / I have constantly monitored myself whether I consume sufficient portions of fruit and vegetable”, and “I had my exercise intention often on my mind / I had my fruit and vegetable consumption intention often on my mind”. Answers can be given on a VAS-scale ranging from do not agree at all to agree completely.

#### Health Outcomes

##### Body Mass Index

BMI will be calculated using the formula “BMI=weight (kg) / height (m^2^)”. Body weight and body height will be self-reported by the participants.

##### Depression

The level of depression will be measured using the Chinese translated Center for Epidemiologic Studies Short Depression Scale (18, 28). Participants will be asked with the stem “In the past week …” followed by 10 items such as “… I was bothered by things that usually don't bother me”, “…I had trouble keeping my mind on what I was doing”, “I felt depressed”. Answers can be given on a 4-point Likert scale ranging from rarely or none of the time (< 1 day) to most or all of the time (5–7 days).

##### Quality of Life

The perceived quality of life will be measured by the Chinese short version of the WHO Quality of Life-BREF (18, 28). The general quality of life will be measured *via* two questions: “How would you rate your quality of life” with an answer category from very poor to very good and “How satisfied are you with your health status” with an answer category from very dissatisfied to very satisfied. The physical health subdomain with seven items will also be used such as “to what extent do you feel that physical pain prevents you from doing what you need to do?”, “do you have enough energy for everyday life”, and “how satisfied are you with your ability to perform your daily living activities?”.

#### Demographic Information

Demographic information will include gender, age, living place, level of education (primary school, secondary school, high school, University or above), relationship status (single or in a relationship), and employment status (fulltime job, part-time job, in training, unemployed/searching job, in pension).

#### Data Analysis

The data will be initially processed for missing values using the multiple imputation method. After examining the distribution of the data, skewed data will be log-transformed and replaced with median values (inter-quartile range). All analyses will be performed using full intention-to-treat analyses, with scores on dependent variables for dropouts carried over using the multiple imputation method. The independent samples *t*-tests, Chi-square tests and F-tests will be used to analyze differences between dropouts and completers regarding baseline characteristics. A 5% level (two-tailed) will be used as the statistical significance cutoff point.

To test the effectiveness of WeChat mini-program-based interventions, linear mixed models with maximum likelihood estimation and generalized linear mixed models with weighted least square estimation will be performed. Regression estimates will be adjusted for participant differences in the number of observations contributing to the mixed models and for variances within subjects (36). Although gender and age will be balanced at baseline, the interaction effects of these variables and treatment should not be omitted (37, 38). Therefore, fixed effects of the linear mixed models will include tests for time (T1-T3) and treatment (IG and WLCG) effects, adjusted for baseline values, gender and age. In addition, −2 log likelihood, Akaike and Bayesian information criteria will be used to assess the model fit.

#### Time Schedule of the Study

**Figure 4:**
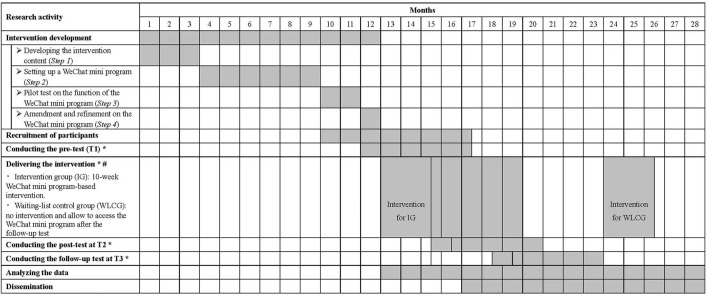
Gantt chart of research activities. *: The lengths of outcome tests at each time point, interventions and follow-ups will be 1 month, 2.5 months (10 weeks) and 3 months, respectively. As the participant recruitment will last for 5.5 months, 4.5 more months were added in each activity. #: The time schedule of intervention delivery in this figure is appropriate for participants recruited in the 12th month only. The time schedule for those recruited in the following 4.5 months will be extended accordingly.

The proposed study will be completed in 28 months between 01 Sept. 2020 and 31 Dec 2022, as shown in Figure 4.

## Discussion

The results of this study will be significant for the future development of online-based aftercare rehabilitation programs for cardiovascular patients in home settings and the development of online interventions in general. Although there are numerous pieces of evidence showing the positive effect of adequate PA and FVC for individuals undergoing rehabilitation (39, 40), many patients fail to maintain lifestyle changes over a longer time frame (41). To transfer the rehabilitation learning outcomes into daily life and to tackle unhealthy habits after discharge from hospital, effective extended rehabilitation and aftercare even following the rehabilitation is necessary.

Considering this, the current study will develop and prove a WeChat mini program-based intervention for PA and FVC promotion in daily life by examining its effectiveness in a sample of Chinese cardiovascular patients in home-based rehabilitation. The potential reach of a WeChat mini program-based intervention is great as it could easily be expanded to reach many WeChat users at a low cost. This can especially be beneficial for those WeChat users who are unable to attend ambulatory or stationary rehabilitation treatment, for instance, due to pandemic restrictions or because of remote living. Furthermore, the theory-based and individualized health behavior change intervention approach in this study constitute the key strategies for designing an effective and comprehensive e-health intervention, which was emphasized in a recent systematic review and meta-analysis (42).

It will be expected that participants in the intervention group will increase their level of PA, FVC and are more likely to adopt a healthy lifestyle. They will also improve their psychological resources of health behavior change and gain more health benefits (normal BMI, decreased depression level, and higher level of quality of life) compared to the control group. In addition, the impact of the current study findings will be maximized by dissemination to key academic, policy, and practitioner groups.

Suggestions for further research range from comparing this WeChat intervention to more traditional interventions and comparing the effects in different regions or countries. However, this is the first important step with determining the main effects regarding behavioral changes, lifestyle adoption, improvements in social-cognitive variables, and health outcomes. However, it is crucial and necessary to identify the key predicting variables for specific behavior changes which needs to be investigated in further research.

## Ethics Statement

The studies involving human participants were reviewed and approved by Research Ethics Committee (REC) of Hong Kong Baptist University (FRG2/17-18/099). The patients/participants provided their written informed consent to participate in this study. Written informed consent was obtained from the individual(s) for the publication of any potentially identifiable images or data included in this article.

## Author Contributions

YD, XL, LG, WL, BS, and SL were involved in the design of the study. YD, LG, and WL were involved in the implementation of the study. YD, WL, and BS drafted the manuscript of the study protocol. All authors read and approved the final manuscript.

## Funding

The current study was supported by the Faculty Research Grant from Hong Kong Baptist University in Hong Kong (FRG2/17-18/099).

## Conflict of Interest

The authors declare that the research was conducted in the absence of any commercial or financial relationships that could be construed as a potential conflict of interest. The reviewer JW declared a past collaboration with one of the authors SL to the handling editor.

## Publisher's Note

All claims expressed in this article are solely those of the authors and do not necessarily represent those of their affiliated organizations, or those of the publisher, the editors and the reviewers. Any product that may be evaluated in this article, or claim that may be made by its manufacturer, is not guaranteed or endorsed by the publisher.

## Abbreviations

CVD: Cardiovascular Disease
PA: Physical Activity
FVC: Fruit-Vegetable Consumption
HAPA: Health Action Process Approach
IG: Intervention Group
WLCG: Waiting-list Control Group
VAS: Visual Analog Scale
BMI: Body Mass Index
WHO: World Health Organization.
